# Exploring the characteristics of capable healthcare professionals

**DOI:** 10.4102/sajp.v71i1.247

**Published:** 2015-07-01

**Authors:** Michael Rowe

**Affiliations:** 1 Department of Physiotherapy, University of the Western Cape, South Africa

## Abstract

**Background:**

In order to negotiate complex clinical environments successfully, healthcare professionals must be more than competent – they must demonstrate an ability to adapt to dynamic situations and continually improve performance. However, emphasis on knowledge and skills alone may ignore opportunities for professional development that enables students to become practitioners.

**Objectives:**

The aim of this study was to ascertain what clinical experts believe are the essential characteristics of capable healthcare professionals that go beyond academic and technical competencies.

**Method:**

This study used a nominal group technique (NGT) to gather data from a panel of South African and international clinical experts, using open-ended questions and qualitative data analysis to determine emergent themes. The NGT is an interpretive research method that can be used to explore poorly defined topics, in this case to develop an understanding of the characteristics of health professionals that go beyond knowledge and technical skill.

**Results:**

Panel members highlighted the importance of knowledge and skills for competent practice, but also explored other aspects of the learning process as part of developing professional identity. These included self-evaluation as part of professional development, building relationships with patients, and the importance of acknowledging students’ emotional responses to the clinical environment. Panel members also discussed the challenge of inappropriate role-models in the clinical context, who may have a negative influence on the development of professional practice.

**Conclusion:**

By including the concept of capability alongside competency in undergraduate healthcare curricula clinical educators can help students develop a sense of being that emphasises professional growth alongside knowledge and technical skills.

## Introduction

### Key focus

#### Background

Clinical reasoning is an intuitive pattern of thinking below the threshold of conscious thought, as well as a rational thinking pattern that occurs deliberately, using information and rules that are acquired through learning (Pelaccia *et al.* 2011). This reasoning process requires an ability to work within the dynamic, non-linear and complex environments of healthcare systems with ill-structured problems that have no clear solutions (Bleakley 2010). In order to successfully manage patients in these complex environments, healthcare professionals (HCPs) must be more than simply competent: they must also demonstrate *capability.* Capability is the extent to which they can adapt to change, generate new knowledge, and continually improve their performance. In addition, they should have positive attitudes towards continuing professional development and lifelong learning, evidence-based practice, information and knowledge management and interprofessional collaboration (Fraser & Greenhalgh 2001). These characteristics describe an HCP who displays a range of characteristics that go beyond simply ‘having’ knowledge and skills.

In order to determine what it means to be an HCP, different approaches have been used to describe what a health professional should be. The CanMEDS framework is one such approach, which describes the health professional as having multiple roles that include a range of competencies within each role (Frank & Danhoff 2007). The relationships between the roles help to inform our understanding of what we understand a health professional to be (Cooke, Irby & O’Brien 2010). A traditional approach to the education of HCPs emphasises the development of these competencies, which usually involves the student performing familiar tasks in familiar environments. This approach enables the assessor to determine what students and HCPs know or are able to do (Fraser & Greenhalgh 2001).

One of the challenges when using competencies to determine what it means to be a health professional is that it can oversimplify the role of the practitioner (Jarvis-Selinger, Pratt & Regehr 2012). Familiarisation with the culture and ways of belonging within a community of practice can form the foundation for a cultural literacy that enables effective professional socialisation (Bleakley 2010; Jarvis-Selinger *et al.* 2012; Paul, Gilbert & Remedios 2013). Therefore practice knowledge is not only the application of skill sets but also participation in the language of practice (Webb, Fawns & Harre 2009).

Health professional educators need to make explicit the rules and norms of thinking and acting based on a value system that is embedded within the profession (Webb *et al.* 2009). There is thus a need to move the health professions education discourse from the current focus on competencies to include a broader emphasis on the relationship between developing knowledge and skills and formation of a professional sense of being during the educational process (Jarvis-Selinger *et al.* 2012).

Including identity formation alongside competency develop­ment in the undergraduate curriculum allows us to reframe our teaching practices toward questions that include a focus on being rather than exclusively on knowing and doing (Jarvis-Selinger *et al.* 2012). The goal is not to replace competencies in the curriculum but to add another dimension to clinical education that extends the focus from doing and knowing to include what it means *to be* an HCP. The aim of this study was to determine what clinical experts believe the essential characteristics of capable HCPs are that go beyond academic and technical competencies.

## Method

### Design

This study used a nominal group technique (NGT) to gather input from domain experts in order to describe what they believed a capable HCP should be, in addition to what they should know and be able to do. The NGT is an interpretive research method that is often used to explore poorly defined topics, such as developing alternative approaches to established programmes or systems (Delbecq, Van de Ven & Gustafson 1975). In this case the goal was to obtain an aggregated description of the characteristics of HCPs that go beyond knowledge and skills. The NGT has the advantage of highlighting the voices of individual clinical experts whilst at the same time allowing a facilitator to aggregate those voices into a single description. It is a useful means of collecting data that can be used to inform decision making around difficult topics (Cohen, Manion & Morrison 2007). There is an element of bias in the technique, in that the panel members are invited to participate in the process and their opinions may already be known to the researcher. For this study a wide variety of experts from a range of clinical disciplines and levels of expertise were included in order to reduce the effect of this potential bias, as well as to develop a comprehensive perspective on the topic.

The 25 panel members included South African and international clinicians and clinical supervisors who were purposively selected as a result of their experience in clinical practice and clinical education (see Table 1). International panel members from the United Kingdom, Sudan and Brazil were invited to participate based on professional relationships with the researcher. Whilst such a large sample is unusual in an NGT study, the comprehensiveness of the participant responses justified the selection and also ensured saturation of responses.

**TABLE 1:** Demographic information from the panel members.

Demographic information	Posts held, years of experience and education	Panel member number
Number of panel members	-	25
Occupation	Professor (academic)	7
	Lecturer (academic)	9
	Clinician	6
	Other	3
Profession	Physiotherapist	11
	Physician	7
	Surgeon	2
	Nurse	2
Years of clinical experience	Range	2–36
	Average	19
Highest degree obtained	BSc	4
	MSc	12
	PhD	4
	MMed	4
	Postdoctorate	1
Additional qualifications	Educational	13
	Clinical	11
	Management	1

*Note:* Panelists did not complete all sections of the questionnaires, hence the totals are inconsistent.

The NGT was run online in 2012, with panellists using a web-based platform to respond to the guiding questions. The facilitator asked the panel what they believe competent and capable HCPs should be, and what challenges exist in developing capable practitioners. Panel members were asked to identify what they thought capable students should ‘be’ in addition to what they were expected ‘to do’. Panellists were asked to reflect on the questions provided and then to complete the online form. In the next stage of the process the researcher anonymised, aggregated and summarised participant responses before sending them back to the panel for individual clarification and comment. The researcher then clustered the responses into categories and asked the panel for their final comments and responses (Cohen *et al.* 2007).

### Data analysis

Data were captured into a spreadsheet directly from the form that panellists completed, which was then downloaded and exported into a text document. The content received from the panel members was analysed qualitatively by the researcher in order to determine themes. Words and phrases were highlighted as being similar or belonging to the same categories, which were then used to determine the emergent themes (Cohen *et al.* 2007). Additional trustworthiness of the data was established using criteria for qualitative research that can be used to determine credibility, transferability and dependability (Cohen *et al.* 2007). The analysis, emergent themes and participant responses were reviewed by two additional researchers who provided critical input to the results and analysis. In addition, the results are presented with quotes from the original text, which serves as supporting evidence for the themes that arose. Together with the critical review of the two other researchers, the quotes serve to establish the credibility and dependability of the claims. The transferability of the claims is limited, considering the specific health and educational contexts that exist in the South African health system.

#### Ethics considerations

The study received ethics clearance from the institutional ethics committee (project registration number 09/8/16). All panel members received an information sheet and provided informed consent to participate. Panellists were not required to participate in this project and non-participation had no negative effects on those who were invited. All responses were anonymous and panellists could withdraw from the study at any stage if they wished and have their responses removed from the database.

## Results and discussion

The results and associated discussion are presented below according to the questions that served to guide the discussion around competent and capable HCPs. These included the importance of developing knowledge and skills as part of competent practice, self-evaluation and professional development, building relationships with patients, and the importance of acknowledging students’ emotional responses to the ethical challenges found in clinical practice.

### Knowledge and skills as part of competent practice

Panellists were asked to describe what they believed clinical competence consists of. They highlighted that a wide range of knowledge (e.g. basic sciences, clinical sciences and health policies) and technical skills (e.g. procedural, communication and interpersonal skills) are essential aspects of clinical competence. The verbatim quotes presented below support the themes that emerged (spelling and grammatical errors made by panel members have been left largely intact; the text in brackets has been added by the author to aid readability):

‘Knowledge of all that informs the [*patient's*] condition. Good interviewing and communication skills. Specific physical examination and clinical reasoning skills to identify the main and associated problems of the patient. Effective treatment, rehabilitation and prevention skills.’

‘Knowledge of basic and clinical sciences. Clinical method knowledge and skills. Communication skills, including listening. Diagnostic ability and therapeutic interventions.’

‘Knowledge and understanding of the body and conditions [*in order to provide*] good care and feedback regarding the patient's condition. Knowledge on assessment and treatment skills are also important. Respect for the profession and the continuous development of skills, knowledge and ethics regarding patient care.’

‘Knowledge of the different pathologies that affect the human body, their medical treatment and prognosis.’

‘Knowledge of the principles underlying the evaluation and treatment of these different conditions. Basic skills in the evaluation and treatment of conditions.’

Panellists noted that competencies (i.e. knowledge and skills) are essential aspects of the curriculum, highlighting what they believed were the knowledge and technical skills that capable HCPs need. These were well aligned with the literature on clinical education, especially in a South African context (Cross 1999; Epstein 2007; Joseph, Hendricks & Frantz 2011). It is clear that for this group of clinical experts a capable HCP must also be competent in terms of possessing the relevant knowledge and skills required for practice.

### Self-evaluation and professional development

Panellists were then asked to describe what makes a *capable* HCP in the context of being able to adapt to a changing environment. Their responses showed that they understood learning to be a personal developmental process, rather than simply the accumulation of facts. They highlighted the importance of self-evaluation as essential to the process of becoming an HCP. The quotes below demonstrate the relevance of self-evaluation as an important aspect of professional development:

‘A capable healthcare professional needs to know when to ask for help. They need to be consciously aware of their areas of weakness or deficits.’

‘Knowing one's strengths and limitations, and a willingness to learn and adapt.’

‘Self-accountability and evaluation of one's own standards of care. Continually measuring processes and outcomes in order to enhance competent performance.’

‘Healthcare professionals should be willing to admit to their own shortcomings.’

The panel members suggested that students should be able to evaluate their own performance, but did not elaborate on the next step, which is the need to seek specific, directed feedback in order to address the problems that they have identified (Boud & Molloy 2013). Students are always generating internal feedback when they work, even in the absence of a teacher. This self-monitoring allows them to identify discrepancies between actual and intended performance, which is determined by their own objectives and what they believe the teacher is looking for (Boud & Molloy 2013). If health professional graduates are to be capable of adapting to dynamic and complex health care systems, they must first learn to identify their own learning needs through a process of self-assessment before they are able to take steps towards improving their practice (Boud & Molloy 2013; Colthart *et al.* 2008). It should be noted that those students who are least able to evaluate their own performance have also been found to be the least competent (Colthart *et al.* 2008). Clinical educators and clinicians who supervise students should therefore aim to help students evaluate their own performance and to follow that up with specific requests for guidance related to the improvement of practice.

### Building relationships with patients

Panellists emphasised that active engagement with patients was a defining characteristic of being a capable therapist. In addition, the nature of the relationship should go further than simply one person providing another with treatment or information. The panel suggested that capable HCPs must be able to relate to patients on a deeper level than a purely technical one:

‘[*Students must*] master their feelings, developing the ability to listen and integrate their knowledge to build a process of care, based in a trusting relationship with patients and their family and with collaborators. Capable also implies being able to connect with the patient on a human level.’

‘The ability to synthesise the evidence and to communicate it to patients and to their families in a fully open manner, … tailoring the care to meet [*the patient's*] preferences or choices, beliefs, and options for self-management.’

‘A caring, knowledgeable person who … takes the time to connect with the patient as a whole person and who listens to the patient. Someone who is not afraid to have difficult conversations about treatment options, pain and death.’

Panellists reported that capable practitioners acknowledge the humanness of their patients, working *with them* rather than simply providing a service. Capable practitioners should therefore aim to build ‘trustful relationships’ with patients, their families and colleagues, developing the social contract between society and the profession by nurturing these qualities in the novice therapist (Hilton 2004). Students must therefore not only acquire the content knowledge and skills of the discipline but also the ‘range of interactional skills specific to the clinical culture, building effective relationships with both colleagues and patients’ (Paul *et al*. 2013:73).

### Emotional context of clinical practice

Panellists were asked to describe their understanding of the role of personal values and beliefs as part of ethical reasoning in capable health professionals. They highlighted the emotional context in which health care is practiced and emphasised the affective components of students’ interactions in the clinical context. They also identified the challenges of authentic, honest engagement with patients:

‘I think that there needs to be more than the mere outward observance of a standard professional ethical code. Emotions play a far greater role in decisions than we are inclined to admit. Emotional maturity, the internalisation of defined values and the habitual, consistent practice of those values is necessary to uphold those values in difficult times.’

‘Ethics in health care starts with the recognition of the conditioning power of disease and of its effects on a human being. Therefore it requires responsibility, empathy with those in sufferance, respect to their autonomy, apprehension of the reality and a clear conviction of our possibilities and limits and above all, of our responsibilities towards the patient.’

‘Having moral courage.’

The role of emotion in ethical practice was emphasised by the panel, who suggested that capable practitioners must consistently put their values into ‘habitual, consistent practice’, instead of the ‘mere outward observance of a standard professional ethical code’. Panellists also reported that the personal values and emotional context of health care strongly support the idea that ethical behaviour is more than an act of doing or knowing, and that it is a state of being. Ethical practice is an inherent part of clinical practice, as clinicians make decisions that are informed by the values, beliefs and emotional factors that are embedded within relationships between themselves and their patients (Edwards, Braunack-Mayer & Jones 2005). There is therefore a strong need for clinicians and clinical educators to develop an awareness of students’ emotional responses to the ethical challenges that exist in the clinical context, and to help students respond to them appropriately.

A lack of moral agency and courage has been identified in HCPs, leading to a negative impact on professional practice (Aultman 2008), so it was gratifying to note that one panel member identified ‘moral courage’ as an important aspect of capable practice. This requires a continuous commitment to and reflection upon personal values and moral behaviours that influence ethical decision making (Clancy 2003; Kidder 2005). Acknowledging students’ emotions may help to facilitate an increased awareness of the ethical dimensions of practice, and therefore decrease the gap between ethical knowledge and ethical practice (Delany *et al.* 2010).

Strategies for developing moral courage include open dialogue about ethical principles and systems, case studies, role modelling by real-life exemplars, and rehearsals in which students practice what they have learned in order to build their skills related to decision making (Kidder 2005; Purtilo 2000).

### Challenges with clinical role-models

In response to the question about the challenges related to developing capability in health professionals, panellists highlighted a lack of positive role-models for students as one of the most important challenges facing students’ professional development. The following quotes are presented in support of the claim:

‘… lack of role models for students in the clinical setting and thus they practice what they see, not what they have been taught. [*They are not*] bold or confident enough to challenge the role-models.’

‘How to maintain their enthusiasm when all around them are losing theirs? How to persuade them that they have the ability to change the system if they don’t like what they find?’

‘Too many practitioners are negative and disempowered and they communicate this to junior members of staff and students (highly unethical as it is corrosive). So ethics is not just about ethical treatment of patients but also of the team around you.’

The panellists’ suggested that those within the system may not always demonstrate appropriate behaviour to students. The influence of clinical role-models on students’ enthusiasm for learning is well established (Ramani & Leinster 2008) and effective clinical teachers must not only have excellent knowledge and skills (Majoor, Ibrahim & Lake 2004) but must also emphasise the psychological and social aspects of health care (Branch & Paranjape 2002). Those responsible for student clinical training should therefore aim to build resilience in students that protects them from the corrupting influence of negative role-models in the health system (Aultman 2008).

Related to this was a concern about students’ ability to challenge the status quo when confronted with challenges to their clinical education. This may be a more general problem in higher education where students are positioned as novices in the teaching and learning relationship and subsequently place teachers in positions of authority (Molloy & Clarke 2005). By virtue of this positioning the student may identify as a passive receiver of information with little sense of responsibility for the learning process and therefore with little confidence to challenge authority. In order to address this lack of agency students should be provided with learning opportunities that help them develop appropriately critical attitudes towards knowledge and authority (Rowe, Bozalek & Frantz 2013). This requires an environment that supports open discussion, the questioning of assumptions and critical evaluation of information, reflection, evidence and argument (Justice *et al.* 2007).

### Limitations

One of the primary limitations of the study is that the NGT is usually carried out in person, with group members in the same physical space. This creates a different environment for discussion whereby subtle nuances of communication are included in the process; these are absent when the discussion takes place online. It cannot be determined if this aspect of the study had an influence on the discussion that took place.

## Conclusion

This article presents the views of a diverse panel of clinicians and clinical supervisors on the characteristics of capable HCPs. The panel reported that capable practitioners should not only have the requisite knowledge and skills as part of competent performance, but should also engage with, and be supported in a process of professional development. This process includes encouraging self-evaluation as part of reflection during learning, creating meaningful relationships with patients, and acknowledging the emotional responses of students in the clinical environment. The challenge for clinical educators and clinicians who supervise students is in understanding the complexity of developing capability in undergraduate and novice health professionals. The importance of role-modelling is underscored as one way in which capable practice can be demonstrated to students, moving the emphasis away from knowledge and technical skills.

Clinicians and clinical educators who work with undergraduate students should consider including some aspects of these findings into their teaching practices, as they help students to develop a sense of identity within their learning and professional community. Capable clinical practice should be described not only in terms of knowledge and technical skills, but should also include the development of a professional identity and a sense of professional being. Students and novice practitioners should be immersed in the language, values and social norms of clinical practice by creating environments that introduce them to the culture of the profession, as well as helping them to develop a sense of moral agency that enables them to challenge the status quo in their learning contexts.
